# Odontogenic tumours in a Chilean population: a retrospective study of 544 cases based on 2022 WHO classification

**DOI:** 10.4317/medoral.26008

**Published:** 2023-10-12

**Authors:** Enrico Escobar, Fernán Gómez-Valenzuela, Cristian Peñafiel, Ana Ortega-Pinto

**Affiliations:** 1 Department of Oral Pathology and Medicine, Faculty of Dentistry, University of Chile, Chile; 2 Department Gynecology, School of Medicine, Pontificia Universidad Católica de Chile, Chile

## Abstract

**Background:**

Odontogenic tumours are infrequent lesions. Studies on the frequency of odontogenic tumours from Latin America are scarce. This work aimed to determine the relative frequency of odontogenic tumours in a Chilean population using the 2022 World Health Organization classification.

**Material and Methods:**

This is a case series retrospective study. We reviewed 35,530 samples from 1975 to 2022 from the Oral Pathology Referral Institute and the Pathological Anatomy Service, Faculty of Dentistry, University of Chile. We utilized the 2022 World Health Organization classification for histological typification.

**Results:**

According to 2022 World Health Organization classification, 544 odontogenic tumours were confirmed. The most frequent odontogenic tumours were: odontoma (*n*=241; 44.3%), ameloblastoma (*n*=109; 20.0%) and cemento-ossifying fibroma (*n*=71; 13.1%). Benign odontogenic tumours corresponded to 538 cases (98.9%) and malignant tumours were only six cases (1.1%).

**Conclusions:**

In our population, odontoma was the most frequent odontogenic tumour followed by ameloblastoma and cemento-ossifying fibroma. Malignant odontogenic tumours were very rare. The results of this study are similar to reports from America, but there are some differences concerning the data from Africa and Asia.

** Key words:**Odontogenic tumours, Chile, keratocyst odontogenic tumour, odontoma, ameloblastoma.

## Introduction

Odontogenic tumours (OTs) constitute a heterogeneous group of lesions derived from the tooth-forming apparatus. They include benign and malignant neoplasms, hamartomas, and cystic lesions with diverse clinical-biological behaviour (1-3). OTs are uncommon, representing significant diagnostic and therapeutic challenges.

In 2005 the World Health Organization (WHO) introduced changes to the OTs classification (1), like incorporating odontogenic keratocyst (OKC) into OTs as keratocyst odontogenic tumour (KCOT). The epidemiological impact of these changes in OTs has been discussed in several comparative studies (4). Later, in 2017, the WHO re-classified OTs incorporating KCOT and calcifying odontogenic cyst into odontogenic cysts (2). Furthermore, cemental ossifying fibroma (COF) was included in the 2017 WHO classification (WHOc) (2). All these changes were maintained in the 2022 WHOc (5). Another change to the 2022 WHOc was the addition of adenoid ameloblastoma (AA) as a variant of ameloblastoma (AME) (5).

The frequency of some OTs is variable and depends on the geographic location and source of diagnosis. Several studies have been carried out in Africa (6), Asia (7-9), North America (4), South America (10,11) and Europe (12) to describe the frequency of OTs. Nevertheless, there are no reports yet using the 2022 WHOc (5). Therefore, this study aimed to determine the relative frequency of OTs in biopsies received at the Faculty of Dentistry of the University of Chile using the 2022 WHOc. Additionally, we compare our series with others reported from different geographical areas.

## Material and Methods

This is a case series retrospective study. We reviewed 35,530 sample records sent to the Oral Pathology Referral Institute and Pathological Anatomy Service, University of Chile, Santiago, Chile. To note, the population of Chile, according to the 2017 census, comprises 17,574,003 people. The clinical records of the cases diagnosed as OTs over 48 years (January 1975 to October 2022) were analyzed by age, sex, and anatomical location of the tumour. The hematoxylin-eosin-stained slides were examined by two oral pathologists (AOP and EE), and the diagnoses were evaluated according to the 2022 WHOc (5). COF were diagnosed including those sporadic lesions located in tooth-bearing areas of the jaws that met the histopathological characteristics of the 2022 WHOc. Cases of COF in people younger than 20 years were more rigorously evaluated to exclude juvenile cemento-ossifying fibromas. For recurrent tumours, the histology of the recurrent and original (primary) lesion were compared, and the original tumour was considered a single case. The jaws were divided into three areas: anterior, premolar, and molar. For cases in the mandible, the molar area included the angle and the ascending ramus. For the different tumours analyzed, relative frequency was recorded, and the age was analyzed using means, median and age range. The present series of OTs classified according to 2022 WHOc were compared with series of OTs based on the 2017 WHOc. Representative series from different regions of the world were selected, when there were several from the same region, those with the largest number of cases were chosen. Due to the recentness of the 2022 WHOc, no series based on this classification are available.

This study was conducted in compliance with the ethical principles for medical research involving human subjects (Helsinki Declaration) and was approved by the Institutional Committee of Ethics of the Faculty of Dentistry of the University of Chile (FIOUCH 13-003) in which the data was anonymized.

## Results

- Pattern of OTs frequency

We found 544 cases of OTs, according to the 2022 WHOc, which represents 1.50% of the total biopsies received in the period. All cases were summarized in Table 1. In the present series, 538 (98.9%) of the OTs were benign, of which 25.9% had epithelial origin, with AME being the most frequent (20%). In the conventional ameloblastoma (CA) group, two desmoplastic ameloblastomas (DA) were included. Mixed OTs corresponded to 45.4%, being odontoma (OD) the most frequent (44.3%). In the 141 complex odontomas (ODX), eight tumours previously diagnosed as ameloblastic fibro-odontoma (AFO) and two as ameloblastic fibrodentinoma (AFD) were included. Meanwhile, mesenchymal OTs corresponded to 27.6 % with the COF as the most frequent (13.1%). Malignant OTs were six cases (1.1%). The most prevalent malignant odontogenic tumour was clear cell odontogenic carcinoma (CCOC) (Table 1).

- Distribution of OTs cases by sex

In this case series a predilection for females (female-male ratio=1.27:1) was observed. The odontogenic tumour (OT) that presented a greater difference in the distribution by sex was the COF with a female:male ratio of 3.18:1, followed by odontogenic myxoma/fibromyxoma (OM) 2.67:1 (Table 1).

- Distribution of OTs cases by age

Table 2 illustrates the distribution of OTs by decade. It was recognized a higher frequency of OTs in the first four decades, with a marked peak in the second decade. OTs such as adenomatoid odontogenic tumour (AOT), OD, OM, and unicystic ameloblastoma (AU) presented a high percentage of cases in the second decade. Interestingly, COF reported a high frequency of cases in the fourth decade (35.2%). The calcifying epithelial odontogenic tumour (CEOT) occurred in the sixth and seventh decades. Finally, the reduced number of malignant OTs did not clarify a characteristic pattern according to the decade of life.

- Distribution of OTs cases by anatomic location

Table 3 shows the distribution of OTs according to the anatomical location. We noted a predominant mandibular compromise, particularly for UA, ameloblastic fibroma (AF), CA, and COF. Nevertheless, peripheral ameloblastoma (PA), central odontogenic fibroma and compound odontoma (ODP) exhibited a prominent maxillary compromise. Last, we did not recognize a localization pattern for malignant OTs.

**Table 1:**
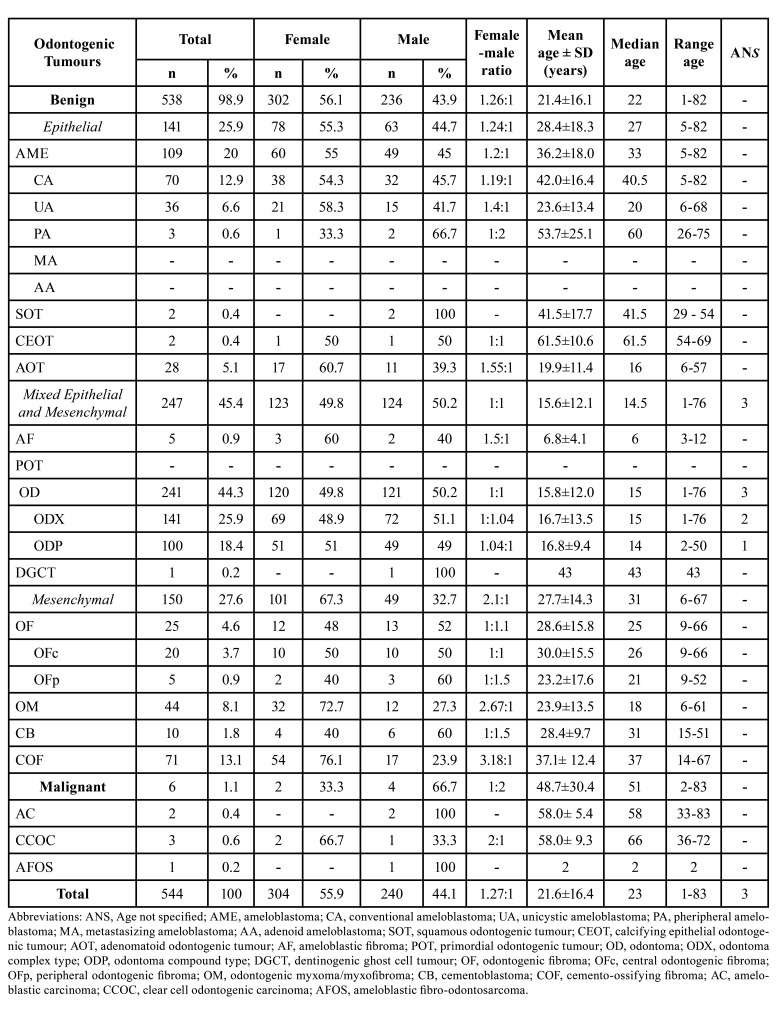
Frequency, sex, and age distribution of odontogenic tumours listed by diagnostic type, according to 2022 WHO classification.

**Table 2:**
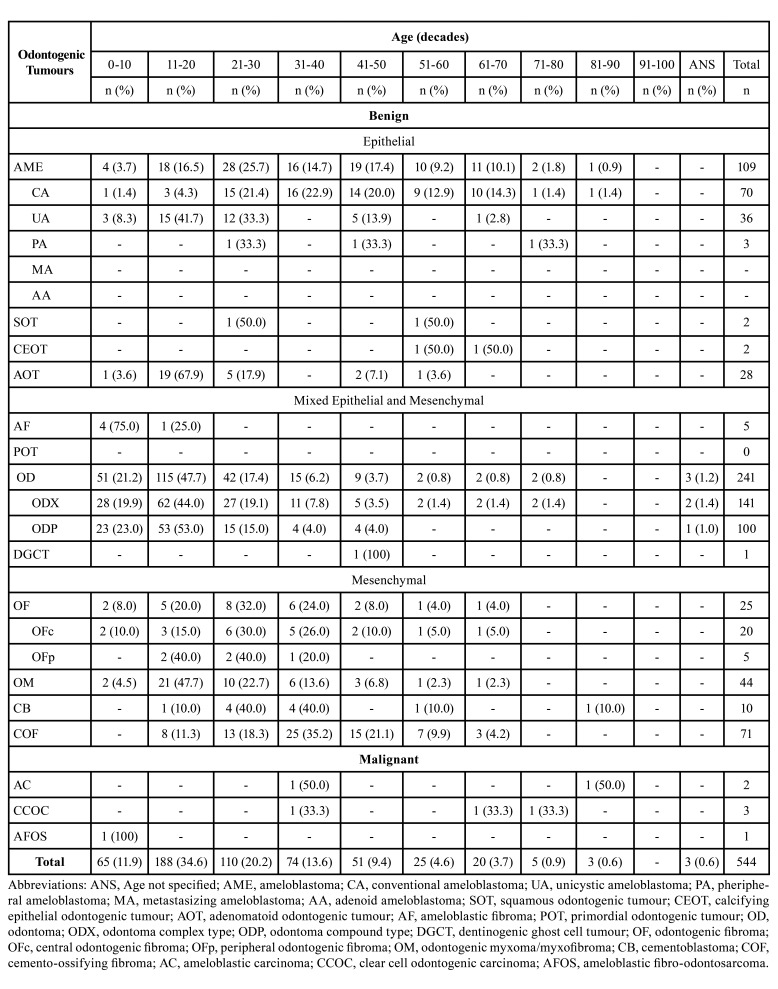
Age distribution of odontogenic tumours listed by diagnostic type, according to 2022 WHO classification.

**Table 3:**
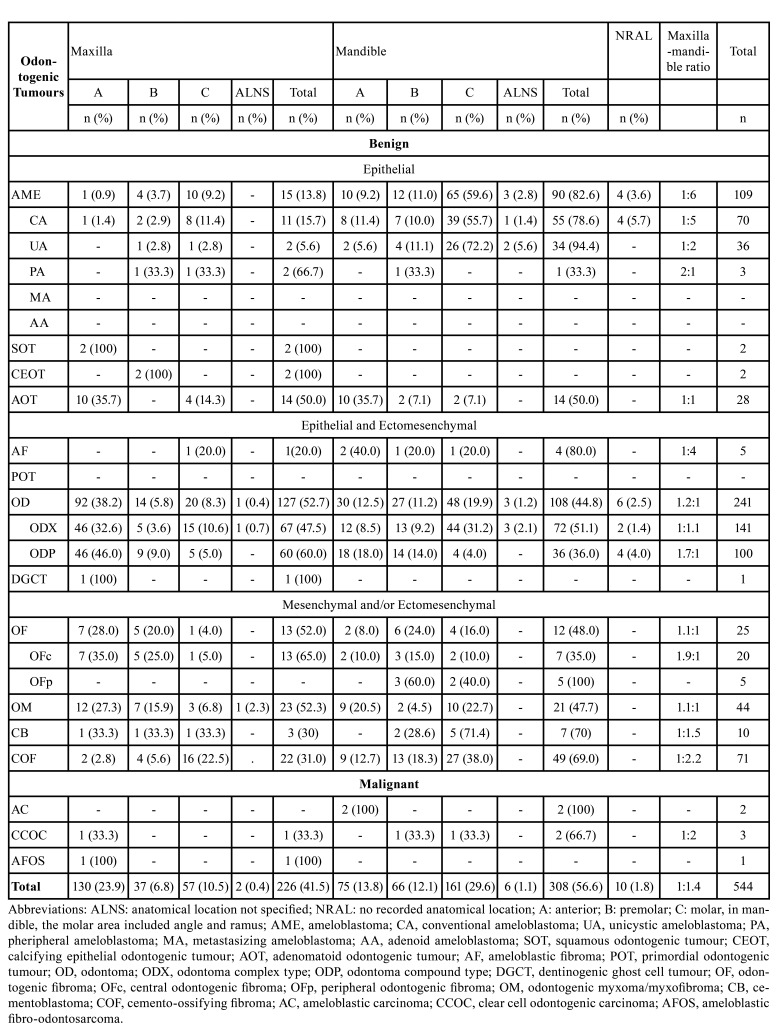
Distribution of odontogenic tumours by anatomic location according 2022 WHO classification.

**Table 4:**
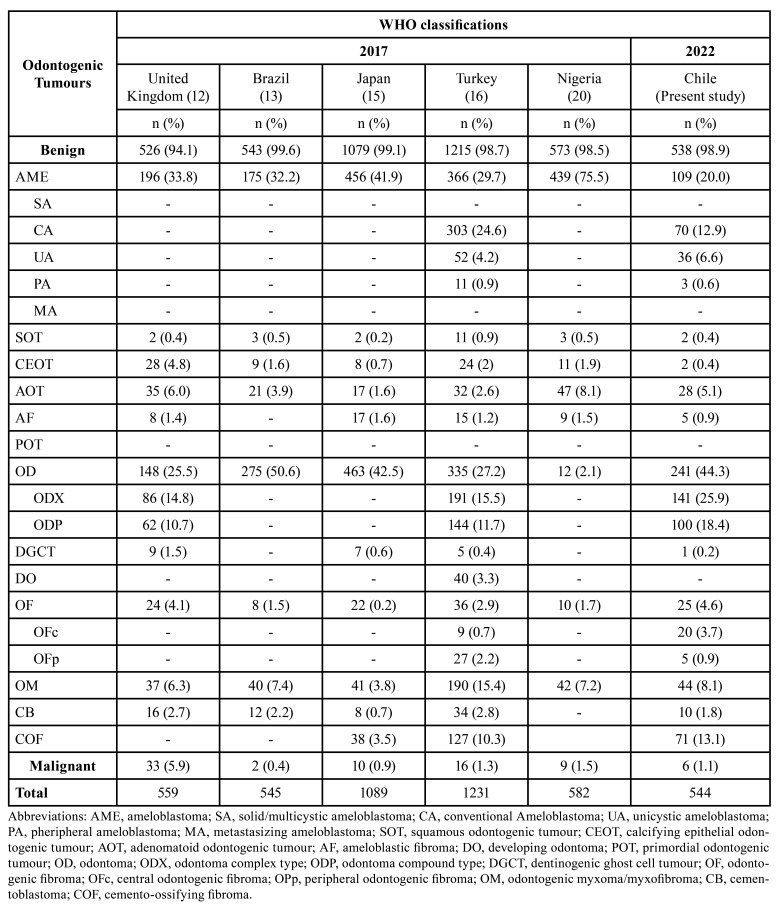
Geographic variation in percentages of odontogenic tumours according to 2017 and 2022 WHO classifications.

**Figure 1:**
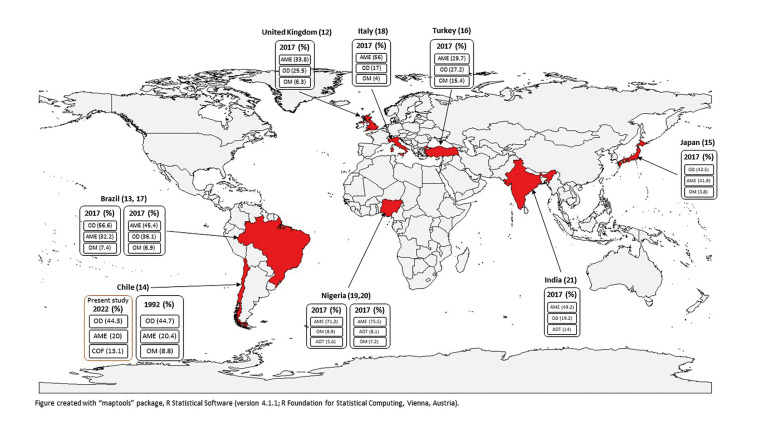
Representation of the worldwide distribution of the three most prevalent odontogenic tumours, categorized according to year of the World Health Organization histological classification used in each series. (%) percentage.

- Geographic variation of OTs according to 2017 and 2022 WHO classifications

The frequencies for OTs according to geographic location for selected studies are shown in Table 4, and Fig. 1 summarizes these comparisons. Of these, a study from Brazil (13) presented the highest percentage of benign OTs, and the series with the highest percentage of malignant OTs was from the United Kingdom (12).

## Discussion

This work represents the second (14) study of the relative frequency of OTs in a Chilean population and includes 544 cases for 2022 WHOc.

In our series, the most frequent OTs were OD, AME, and COF. OD was also reported as the most frequent OT in Israel (13), Pelotas/São Paulo (Brazil) (13), Finland (13), and Japan (15). On the other hand, series from United Kingdom (12), Istanbul (Turkey) (16), Northeast of Brazil (17), Marches (Italy) (18), Northern and Southern Nigeria (19), Southern Nigeria (20), and India (21), reported AME as the most frequent OTs using 2017 WHOc. Interestingly, other studies with 2005 WHOc, which includes KCOT, reported AME as the most frequent OTs: Ibadan (Nigeria) (6), Sichuan (China) (7), Sri Lanka (9), Kayseri (Turkey) (22), and Malaysia (23).

OTs are rare lesions, and it is unclear if the differences between series are due to geographical variations or the type of institution from which the data came from (e.g., dental schools, hospitals, etc.). The vaster knowledge of the genetic-molecular profile that has progressed in recent years will contribute to an accurate understanding of the etiopathogenic mechanisms of OTs (1-5). Furthermore, these mechanisms could clarify whether the differences in the geographic distribution of OTs in the world are associated with genetic and environmental factors.

In the present study, the ages of the patients ranged from one to eighty-three years. Most OTs occurred within the second and third decades, with a peak in the second decade in Uberlandia (Brazil) (10), Pernambuco (Brazil) (11), Japan (15), Northeastern Brazil (17), India (21), Malaysia (23), and Greece (24). Other series showed a peak in the third decade (7-9,19,20,25).

In general, benign OTs displayed a female predilection, like series from Brazil (11,17,25). However, other series reported a preference for males: Sichuan (China) (7), Northern China (8), Marche (Italy) (18), India (21), and Greece (24). Interestingly, cementoblastoma (CB) and odontogenic fibroma (OF) displayed a slight male predilection in our study.

Our study confirmed the mandible as the most affected site, mainly for AME and COF, like other series (7,20,24). Moreover, the posterior area of the mandible was significantly involved in UA, CA, and COF.

Most OTs presented a central location (intraosseous) in the jaws. Nevertheless, we recognized eight cases (1,47%) of peripheral (extra-osseous) OTs (POTs): peripheral odontogenic fibroma (OFp) (five cases) and PA (three cases). In other series, POTs represented between 0.4% to 7.6% of all OTs (9,26), where OFp (6,8,16,26) and PA (6,8,11,12,16-19,21,23,24,26) were the most frequently observed in soft tissues (peripheral). Other POTs reported in the literature included CEOT (11,16,26), squamous odontogenic tumour (27), OM (6,18,19,26), AOT (26), AF (6,18,26), dentinogenic ghost cell tumour (12,16,26), and ameloblastic carcinoma (AC) (9).

Some studies did not report POTs (4,7,10,13,15,20,22,25). It is indispensable to emphasize that POTs are exclusively perimaxillary soft tissue lesions, therefore, it is necessary to exclude the exteriorization of central (intra-osseous) OTs through radiographic studies.

The 2005 WHOc considered four types of AME: solid ameloblastoma (SA), UA, PA and desmoplastic ameloblastoma (DA) (1). However, in 2017 WHOc (2) and 2022 WHOc (5), DA was considered a histological subtype of SA and CA respectively, and metastasizing ameloblastoma (MA) was catalogued as a variant of AME. In 2022 WHOc, AA was considered a new variant of AME (5).

To note, despite differences in clinical behaviour, histomorphology and imaging characteristics, only some series describe the frequency by clinicopathological types of AME (6,8,9,16,17,21,23). In this study, CA and UA represented the most common types, like series from Africa (20), America (17), and Asia (8,9,23). Interestingly, UA corresponded to the second frequency of AME in Africa (6), America (17), Asia (8,9,21,23) and Europe (16). The DA was reported in Northern China (8), Sri Lanka (9), and Malaysia (23). We did not observe cases of AA or MA in our series.

In this study, CA and UA showed a female predilection, however, other studies showed male predilection (8,9,21). Mandible was most affected for CA and UA, especially the posterior area (6,8,9,21). Lastly, cases of CA were distributed among the third and sixth decades, while UA occurred between the second and third decades, with a peak in the second decade. Other studies described a rise in the second (6), third (9,21), and fourth decades (8) for CA, and a peak in the second (6,8,9,23) and third (21) decades for UA.

OD were the most frequent OTs in this Chilean 2022 WHOc series (43.3%). Despite the high frequency of OD, other series present a lower frequency in 2005 WHOc (7,23,24,26) and 2017 WHOc (19,20). OD frequency could be underreported because of its scant symptomatology, small size and slow and self-limited growth so surgical excision is not performed (9). OD exhibits two histological types: ODP and ODX. However, in some series the ODs were examined differentially (7,8,10,12,16,22). The 2022 WHOc considers both types of odontomas as a unique entity.

Females were slightly more affected than males for ODP, as in other series (7,8,22). In contrast, ODX presented a slightly male predilection, like various reports (7,8,12,16). Both ODX and ODP showed a peak in the second decade, like series from Northern China (8). Other studies showed a predilection for ODP in the second decade (7,22). In contrast, various reports showed an ODX predilection for third (7) and fourth (22) decades. ODP was predominantly observed in the anterior maxilla (46.0%), like in Northern China (8), Istanbul (Turkey) (16). However, other studies reported a high frequency in the mandible (16,22). Most ODX were mainly located in the anterior maxilla (35.0%) and posterior mandible (29.0%), like Sichuan (China) (7), Northern China (8), Istanbul (Turkey) (16), Kayseri (Turkey) (22). Only one study did not detect cases of OD (6). This could be due to the fact that the OD are not detected clinically or radiographically, are not surgically removed, or the samples are not sent for histopathological study. In the 2005 WHO classification, AFO was considered an independent tumour, and AFD was included in the spectrum of AF (1). Interestingly, 2017 WHOc (2) and 2022 WHOc (5) included these lesions as developing OD. The exclusion of AFO and AFD from the 2017 WHOc and 2022 WHOc has generated disagreements (12), especially for AFO, because of clinical evidence of expansive and osteolytic growth (28). Hence, some of these lesions could be compatible with true neoplasia, meanwhile, other lesions suggestive of AFO and AFD would correspond to hamartomatous lesions (28).

In our study, COF ranked third (13,1%) for 2022 WHOc. Previously, COF was excluded from 1992 WHOc (29), however in 2005 WHOc (1), it was included in the group of bone-related lesions as a fibro-osseous lesion. In 2017 WHOc (2) COF was typified as a benign mesenchymal OT. Furthermore, in 2022 WHOc (5), its inclusion in this OTs group was maintained. Odontogenic COF must be separated from non-odontogenic variants such as the non-odontogenic juvenile trabecular and psammomatoid types, especially those of extragnathic localization in facial bones, and rapid growth in children and adolescents (2,17). In this sense, localization in tooth bearing region of jaws is considered one of the diagnostic criteria for odontogenic COF (5). Most cases occurred in females, in the fourth decade, and in the mandible. Previous series reported a similar pattern: females (15,16,19,21), within the fourth decade (15,19), and located in the mandible (15,16,18).

In our series, OM ranked fourth according to the 2022 WHOc (8.1%). Most cases occurred in females, in the second decade, with a slight preference for the maxilla. Most of the series exhibited principal affection for females (8,10,19,20). Nevertheless, some studies showed a predilection for males (6,22,26), while others did not recognize differences (9,18). For age distribution, some series reported initial diagnosis of OM in the second (9,10,22) and third decades (6,9,17,24,25). For anatomic location, the maxilla was likewise the most affected site in various series (8,9,17,24). In contrast, some studies reported mainly mandibular compromise (6,7,12,15,17,19-21,26).

AOT ranked fifth in our series. In 2005 WHOc (1), AOT was reclassified as a benign OT derived from odontogenic epithelium but without odontogenic ectomesenchyme (3). Moreover, 2017 WHOc (2) and 2022 WHOc (5) confirmed the exclusive epithelial lineage of AOT based on calcification foci that would correspond to a failed secretion attempt of the enamel matrix. In our series, AOT occurred mainly in the second decade and anterior area of the maxilla and the mandible. These results agree with previous reports for maxilla (8,9,17,21,25) and mandible (7,8,22).

In our series, we reported few malignant OTs: CCOC (three cases), AC (two cases), and ameloblastic fibro-odontosarcoma (one case). Our results were similar to those reported in Pelotas/São Paulo (Brazil) (13), Finland (13), Japan (15), Istanbul (Turkey) (16), Northeastern Brazil (17), Southern Nigeria(20), and India (21). Regarding the group of malignant OTs, the reports with the highest percentages were around 6% of the total cases and some of these include referred cases (8,12,22). Most of the malignant OTs reported in the literature corresponded to carcinomas, which included AC (6-8,10,22,25,26), primary intraosseous squamous cell carcinoma (PIOSCC) (6-8,10,12,15,16,19,20,22,23), CCOC (7-13,16-18,22,23), ghost cell odontogenic carcinoma (7,8,12), PIOSCC arising from OKC (9), PIOSCC arising from odontogenic cysts (10), odontogenic carcinosarcoma (12,16), and sclerosing odontogenic carcinoma (12). In addition, reports for odontogenic sarcomas are scarce and include ameloblastic fibrosarcoma (6-9,21), ameloblastic fibrodentinosarcoma (11), and odontogenic sarcoma (13,15,16,18-20).

When comparing the diverse OTs series, the frequency of OTs in Chile was similar to reports based in the 2005 WHOc from Kayseri (Turkey) (22), and Greece (24). In contrast, some series reported a frequency of OTs greater than our study: Mexico (4), Pernambuco (Brazil) (11), Malaysia (23), and Ceará (Brazil) (25). In addition, various series using the 2017 WHOc reported higher frequency for OTs: Istanbul (Turkey) (16), Northeastern Brazil (17), and India (21). Nevertheless, series from Japan (15) reported a similar frequency (1.8%) to our findings. Our work confirmed that most OTs are benign, constituting 98.9%. These results are in accordance to studies in which benign OTs represented between 94.1% (9) and 99.2% (21) according to 2017 WHOc. Instead, the frequency of malignant OTs in our study was low and represented 1.1% of OTs, which is equally consistent with most studies.

It is interesting to note that the variations in frequency may be due to the few signs and symptoms manifested by asymptomatic slow-growing benign OTs. Therefore, these lesions are not detected clinically and are not included in the series of OTs (18). In contrast, benign OTs of local aggressiveness with greater clinical manifestations, such as AME and OM, would be detected more frequently. For the same reason, malignant OTs are also likely to be diagnosed in most affected patients due to their rapid growth and sometimes pain and paresthesia. This cause can lead to the notification of more aggressive OTs. In the same way, for this study, another cause of variation in the frequencies is that the samples are diagnosed in an Anatomic Pathology Service of a School of Dentistry, in which most of the biopsies are sent by dentists, so there could be differences with series from Anatomic Pathology Services of General Hospitals, in which biopsies are sent by both physicians and dentists (15). It is also important to consider that some biopsies may be referred from other pathology departments and should be reported separately in case series (12). Therefore, we recommend making comparisons between studies with similar methodology in terms of the source of the diagnosis for the samples.

When evaluating the frequencies for TOs from our previous report (14) based on 1992 WHOc (29) against the current series, we observe that the relative frequencies for some TOs are similar, especially for OD (44.7% for 1992 WHOc, and 44.3% for 2022 WHOc) and AME (20.4 % for 1992 WHOc and 20%, for 2022 WHOc). However, in the latest editions for classifications of OTs in 2005 WHOc (1), 2017 WHOc (2), and 2022 WHOc (5) critical conceptual changes have occurred. For example, in the 1992 WHOc (29), clear cell odontogenic tumour is classified as a benign epithelial lesion. However, since the third edition of the WHOc (1), it has been considered a malignant lesion called CCOC. Therefore, these lesions are now included in the group of malignant OTs, increasing their frequency from our previous series (0.6%) (14) to the current series (1.1%). Another noTable change was the inclusion of the COF in the 2017 WHOc (2), and maintained in 2022 WHOc (5), which in this series was the third most frequent, displacing the OM, unlike the previous report in Chile, in which the OM was observed in the third frequency (14). It is important to note that the inclusion of COF is not fully consensual (17), and that the inclusion of non-odontogenic entities makes it difficult to compare between series for OTs.

## Conclusions

The present study is one of the largest OTs series in Latin America. Like most OTs series, the cases were principally diagnosed in the second decade and benign OTs were more frequent than malignant OTs. Moreover, like most studies conducted in America, odontoma was the most frequent OT in our population according to 2022 WHOc. Nevertheless, in series from Africa and Asia, the most prevalent OT was AME. In addition, malignant OTs in series from Africa and Asia were more prevalent than in American series.
